# The operations of contemporary Han Chinese privilege

**DOI:** 10.1177/0920203X231193086

**Published:** 2023-08-10

**Authors:** Reza Hasmath

**Affiliations:** 3158 University of Alberta

**Keywords:** ethnic minorities, Han Chinese, privilege discourse, labour market, media, daily interactions

## Abstract

This article discusses the conceptual underpinnings and performance of Han Chinese privilege in the People’s Republic of China. It suggests that Han Chinese privilege has gained salience from specific public policies and philosophies of governance. This is aptly viewed across a range of sites, including the labour market and media, and involves state institutions and micro-level everyday interactions between the Han Chinese and the ethnic minority populations. Finally, the article theorizes why a robust Han Chinese privilege discourse has not emerged, and remains largely an unacknowledged concept.

The literature on ethno-racial privilege has predominantly focused upon the dichotomous relationship between the privileged ‘white’ population and the racialized ‘Other’. This is not altogether surprising given the make-up of ethno-racial hierarchies in the majority of multi-ethnic Western jurisdictions.^1^ Mobilization by ethno-racial groups resulting from continued structural political, social, and economic inequities – evident in the cases of South Africa’s post-apartheid movement, indigenous groups dehumanization in Australia, Canada, or the United States, or ‘Black Lives Matter’ protests in numerous Anglophone Western nations – has garnered significant media attention, helping to crystalize conceptions of white privilege and supremacy in the collective consciousness.^2^ In contrast, issues of ethno-racial disparities and privilege in ‘non-white’ dominant societies have garnered far less public attention. A significant opportunity, thus, exists to add to the analytical conversation by looking at other forms of ethno-racial privilege that is not solely predicated on Western jurisdictions’ experiences.

In this spirit, this article explores the operations of ethno-racial privilege vis-a-vis the world’s largest ethnic group, the Han Chinese. Although a growing body of literature exists on the ethno-racial dynamics at play in the People’s Republic of China (PRC), it has predominantly focused on the experiences of the ethnic minority populations; or, examined the potential discriminatory behaviour among Han Chinese towards specific, marginalized ethnic minority groups (e.g. Hui, Tibetan, and Uyghur).^3^ Absent in much of this research is an explicit analysis of the operations of privileges amongst the Han Chinese, the ethnic majority in mainland China who comprise 91.1 percent of the nation’s 1.4 billion total population.^4^

In the 1980s and 1990s prominent scholarly works conceived of Han Chinese identity as an empty category that primarily defines those who are not one of China’s 55 officially recognized ethnic minority groups.^5^ Similarly, Stevan Harrell contended that gathering and analysing data on Han Chinese as a category is challenging, since Han Chinese identity is generally an ‘unmarked characteristic that can be delineated only in contrast to the ethnic [O]ther’.^6^ While this perspective still has validity in the present day, James Leibold^7^ notes that for many in China – especially those born in the post-market era (1978 onwards) – the category of Han Chinese has increased utility, insofar as it delineates the achievements of specific political, economic, and personal objectives. This suggests that an analytical deep dive into the underpinnings and operations of Han Chinese privilege is paramount not only for understanding contemporary ethnic relations in China, but also for contributing to the broader intellectual conversations about ethno-racial privilege in other jurisdictions.

The article will proceed in fourfold. The first section introduces the conceptual framework of ethno-racial privilege, with notable reference to white privilege as a philosophical underpinning. While the theoretical construction of white privilege is largely based on Western jurisdictions’ experiences, the essential properties of the concept – one predicated on identifying potential societal structures and power and their underlying dynamics – have transformative, analytical utility to dissect Han Chinese privilege. A brief history of ethnicity in contemporary China then follows in the second section. It will be demonstrated that many facets of Han Chinese privilege stem from specific public policies and philosophies of governance. This is aptly seen in the third section where manifestations of Han Chinese privilege can be viewed across a range of areas, including the labour market and media, and involves state institutions and micro-level daily interactions between the Han Chinese and non-Han Chinese populations. Finally, the article theorizes why a robust Han Chinese privilege discourse has not emerged, and remains largely an unacknowledged concept.

## The ethno-racial privilege framework

The ethno-racial privilege framework is dominated in the academic literature by conceptualizations about the philosophical essence, and performative nature, of white privilege. For scholars such as Ruth Frankenberg, ‘whiteness’ is ‘a location of structural advantage of ethno-racial privilege’.^8^ That is, white privilege derives from political, economic, and social structures where resources and power favour the white group in relation to the non-white group.^9^ Moreover, whiteness is inherently unstable and in flux, constantly being constructed, reconstructed, and contested from within, and outside, the group. While skin pigmentation typically serves as one of the dominant criteria for ascribing ethno-racial identity, criteria such as language, religion and culture also play a role in this process.^10^ For instance, Polish migrants in the United Kingdom, or Irish migrants in the early history of the United States, although being phenotypically white, both experienced racialization and exclusion.^11^

Ethno-racial identity, and consequently, ethno-racial privilege, is inherently intersectional. It is influenced by multiple interconnected subject positioning, including most prominently, age, gender, class and religion. John Hartigan’s research found that even non-migrant whites may be marginalized by being viewed as rednecks and white trash; they exist on the periphery of the construction of whiteness.^12^ Thus, the privilege afforded to those constructed as white may be amplified or decreased resulting from other ascribed social positions. Jacqueline Housel goes so far as to suggest that ‘being white, then, does not ensure access to white privilege’.^13^

Ethno-racial privilege, in this context, is defined as the added benefits awarded throughout one’s life course due to being a member (or perceived as such) of a dominant ethno-racial group, for example white or ‘white-passing’. Over time, these advantages become expected by, and for, the group. That is to say, individuals within the group perceive the benefits afforded to them as natural. This privilege is granted even if the individual is unaware of the benefits provided to them. Peggy McIntosh, for instance, recognizing the propensity of whites to be unaware of their own privilege, wrote a detailed list of the advantages that she experienced because of her whiteness.^14^ These include being able to easily match a bandage to one’s skin tone, not being racially profiled while shopping, and, if desired, the possibility to associate with people of her own race most of the time. Scholarship on white privilege has unanimously suggested that the advantages afforded to whites over non-whites can be recognized in almost every facet of society, including, but not limited to, romantic relationships, the labour market, extracurricular social engagement, health care, pop culture representations, all levels of education, neighbourhood quality, access to beauty products, and the availability of cultural foods. Scholars such David Roediger have talked about the psychological ‘wages of whiteness’ when it comes to understanding white privilege, wherein segments of the white community that do not necessarily enjoy the material benefits of whiteness still receive psychological and emotional benefits of knowing that they are not non-white.^15^

Overall, these micro-level everyday realities point to the underlying privilege of whiteness being the societal norm.^16^ That is, the white population can reasonably expect to progress through life knowing they will be prioritized by the government, market, and civil society. Non-whites, in contrast, exist on the margins. While their needs may be addressed, they are not the first priority. In many instances, non-whites must mobilize, engaging in collective action and other forms of resistance, to have their basic needs addressed, and/or to gain access to the same rights and privileges extended to the white population.

Scholars have also focused on bringing attention to the reasons why such ethno-racial privileges exist. The general consensus is that ethno-racial privilege in Western jurisdictions is largely rooted in past acts committed by whites such as slavery, colonization, and imperialism.^17^ Consequently, white privilege has gained a certain innocence to it, in that the current-day white population are not responsible for such past injustices and therefore, are not responsible for their privilege. This view is inherently problematic. Foremost, it is counterfactual to the mechanism that maintains white privilege. White privilege was not just a result of discrete historical events, but rather, it is contingent on the continued exploitation of the resources and labour of non-whites. Moreover, this domination is institutionalized and in part, sustained through the adoption of past and present public policies in healthcare, education, housing, policing, taxation, that continue this exploitation. Zeus Leonardo picks up this point and argues that white domination emerges ‘out of a patterned and enduring treatment of social groups . . . it is secured through a series of actions, the ontological meaning of which is not always transparent to its subjects’.^18^

Anne Bonds and Joshua Inwood reach similar conclusions in their discussion of ethno-racial supremacy.^19^ They contend that the material and hegemonic advantages afforded to whites exist as a direct result of the construction and maintenance of ethno-racial structures that on the one hand, cause violence for non-white ethnic minorities, and on the other, benefit whites. Hence, white supremacy is not a past reality, nor is it merely an ethos espoused by extremist groups. Rather, it is an accurate description of the current reality within white-dominated states. Further, the everyday logics of this system ‘are reproduced through spectacular and mundane violences that reaffirm empire and the economic, social, cultural and political power of white racial identities’.^20^

The insights gained from the ethno-racial privilege literature, dominated by the white privilege discourse, provides an entry point to better understand the operations and performance of Han Chinese privilege. It allows for the development of an analytical baseline of the essential attributes that are common (and potentially differentiated) in Han Chinese privilege.

## The construction of ethnic identity in China

Han Chinese, as a category, holds considerable commonality with the category of whiteness. Both are comparatively recent social constructions that fused together large subgroups of people, who themselves possessed their own distinct cultures and languages. Although Han Chinese as a defined ethno-racial category did not exist prior to the 20th century, ‘the modern ethnonym builds on a much older historical formulation, one that informs the “cultural stuff” which defines the boundaries, symbols and sentiment of Han today’.^21^ This older formulation gained meaning in the contemporary era through the introduction of conceptions of ethnicity and race by grafting the two together.^22^ Han Chinese identity could be mobilized in a process of opposition and contrast to the ruling Qing Manchu dynasty.^23^ This process is not altogether dissimilar to how the varied European ethnic groups in settler societies such as Australia, Canada, and the United States, became incorporated into a white racial identity, in contrast to the ‘black’ and indigenous populations.^24^

Alternatives to a Han Chinese centric approach quickly emerged in the post-Qing Chinese republic, largely out of practical necessity, in that essential border regions were the homelands of Tibetans, Uyghurs, Mongolians, and other minority groups. As such, a more pluralistic vision of a ‘Republic of Five Peoples’ – Han, Manchu, Mongolia, Tibetan, and Hui (which generally referred to all Muslim ethnic groups) – was proposed by Republican leaders. When Chiang Kai-Shek took over as leader of the Republic of China, he rejected this approach and instead advanced the concept of a single-race republic, in which the Republic of Five Peoples belonged to a supra-ethnic that had existed since time immemorial.^25^ All of these aforementioned conceptions of Chinese identity continue to influence the ethno-racial discourse in contemporary mainland China.^26^

As a Han Chinese-dominated party, the Communist Party of China (CCP) had to develop a strategy to manage the minority-heavy bordering areas of northwest China and Inner Mongolia. At minimum – and arguably perhaps at the level of rhetoric – the CCP initially sought to protect the minority border populations from Han Chinese domination. There are three primary reasons for this. First, they drew inspiration from the Soviet Union’s ethnic management policies. Second, throughout the Long March (1934–5) Chinese communist leaders encountered the ethno-cultural diversity within China as they travelled from southeast to the northwest. Furthermore, they offered ethnic minorities preferential treatment, official recognition, and the promise of autonomous regions, in exchange for their support against the Kuomintang during the Chinese Civil War (1927–37; 1945–9), and against the Japanese during the Second Sino-Japanese War (1937–45). Third, the Communists adopted their position out of a desire to oppose and stand in contrast to the Kuomintang in almost all areas.^27^ Notwithstanding these reasons, in the end, the CCP rejected the Soviet Union’s policies of ethno-federalism, and took a position closer to the Kuomintang stressing the ‘inclusiveness’ of the Han-dominated Chinese nation.^28^

In 1953, the CCP encountered a problem: no one was entirely sure who an ethnic minority was, nor was anyone sure how populous these groups were. Thus, the state undertook the considerable task of categorizing the ethnic population. At first, they employed a census which catalogued demographic data, as well as respondents self-reported ethnicity. This preliminary effort identified over 400 ethnic groups, a situation that was both surprising and unworkable from the state’s perspective. In 1954, the CCP dispatched teams comprised of ethnologists, linguists, and party cadres to regions with substantial ethnic minority populations with the goal of categorizing and identifying prospective ethnic groups. The teams were not only interested in the characteristics – culture, religion, and social history – of the various communities, but also in the perceived feasibility of merging community subgroups into new ethnic groups. This approach was successful in shrinking the previous 400+ ethnic groups identified through self-identification, into 39 ethnic groups, who subsequently gained official recognition. An additional 16 ethnic groups gained official recognition in 1965, with the last ethnic group added in 1979.

Following the recognition of the initial 39 ethnic minority groups, the state enacted a regional ethnic autonomy policy, which introduced a series of affirmative action policies for ethnic minorities. The state expanded this affirmative action regime in 1984 and 2001.^29^ Ostensibly, the purpose of these policies was to improve national unity, and to ‘modernize’ and ‘improve the economic conditions’ of the ethnic minority populations. In effect, the CCP constructed the image of ethnic minorities as ‘backward’, in contrast to Han Chinese who were depicted as ‘vanguards’ of the revolution, and the most advanced in a Marxian conception of progress.^30^

Ironically, whites in Western Europe, Australia, and North America invoked similar notions of progress and backwardness to justify colonialism, indigenous/Aboriginal oppression, and/or foreign intervention. Nonetheless, there existed a certain contradictory attitude by CCP leaders who saw themselves as both essential to safeguarding the revolution, and as a threat to national stability and unity.^31^ Mao Zedong himself warned in a 1953 directive to the party that ‘Han chauvinism’ was ubiquitous among the party and populace writ large, and that they had to eliminate it.^32^ In effect, ethnocentrism was contradictory to the founding principles of the PRC. But paradoxically, as then Premier Zhou Enlai pointed out, the various minority groups must ‘secure the help of the Han Chinese and more advanced nationalities, study the experiences gained by these nationalities, and induce their own cadres to work for the good of their nationality’.^33^Affirmative action policies came to an abrupt halt during the tumultuous climate of the Cultural Revolution (1966–76). Extremists within the CCP attacked ethnic minority populations for their traditional practices, and pressure to assimilate was exerted upon minorities.^34^

After the Cultural Revolution ended, many of the prior policies were reinstated, and from the 1980s onwards, in keeping with the general reformist attitude of the time, expanded affirmative action policies for ethnic minorities were introduced.^35^ Although these policies vary by jurisdiction – province, autonomous region, or municipality – they tend to include certain exemptions from the government’s family planning programmes, preferential treatment in school examination and admission, hiring and promotion in the state sector, infrastructure development, and improved access to political office, allowing tax revenues to stay in ethnic minority regions.^36^ It is debatable whether these policies have been effective in achieving their intended goals of improving national unity as well as the economic conditions of ethnic minorities.^37^ Nevertheless, within the National People’s Congress and at the regional level, ethnic minorities have reasonable levels of representation.^38^ Theoretically, nominal political autonomy is afforded to areas in which minorities are geographically concentrated including the five provincial-level autonomous regions of Guangxi, Inner Mongolia, Ningxia, Tibet, and Xinjiang; as well as numerous autonomous cities, prefectures and municipalities. In reality, however, the CCP retains political control over all autonomous jurisdictions, which is vividly evident in present-day Xinjiang and Tibet.^39^

The actual effectiveness of policies to improve the socio-economic conditions of ethnic minorities, and to enhance national unity, can be contested. Although some ethnic minorities now possess comparable, or even higher, earnings than the Han Chinese population,^40^ this is not a universal experience as will be discussed in the subsequent section. Additionally, these policies have caused some individuals to ‘rediscover’ their ethnic identity and have imbued ethnic minority identities with increased significance.^41^ Albeit, there are certain limits to this process of ‘rediscovery’. Unlike most Western multi-ethnic states, in China, ethnic status cannot be altered at one’s own discretion or on the basis of self-identification. Nevertheless, when the CCP slightly modified the rules on ethnic registration, permitting those born of mixed marriages^42^ and those previously ‘miscategorized’ to re-apply,^43^ between 1982 and 1990 ethnic minorities as a total share of the national population rose sharpy from 6.62 percent in 1982 to 8.01 percent in 1990. In fact, the ethnic minority population has almost doubled from 67 million in 1982 to 123 million in 2005.^44^ This is an increase that cannot be accounted for by birth rates alone, and can be largely attributed to the fact that ethnic minority privileges were perceived as an advantage.

Interestingly enough, these privileges have resulted in increased Han Chinese resentment towards the perceived unfair advantages afforded to the ethnic minority populations. This is even to the extent of stating that it is ‘reverse discrimination’ against Han Chinese.^45^ Scholars have also become more critical of the state’s institutionalization of ethnic differences. Ma Rong, for instance, suggests that ethnic policies have led to new hurdles in the construction of a collective national consciousness.^46^ This questioning of the CCP’s official ethnic policies has signalled, and given cover, for Han Chinese netizens to voice their dissatisfaction with the ethnic status quo in China.^47^ Notably, these criticisms do not always attack the CCP. Instead, much of Han Chinese frustration is aimed at ethnic minorities themselves, or those who are perceived as supporting ethnic minorities. Muslim minority groups in China, in particular, are the target of negative comments, often being framed as outgroup members who do not belong in China. Even though those engaged in such practices are not directly supporting a Han Chinese-centric or Han Chinese supremacist viewpoint, by constructing ethnic minorities as ‘foreign’ and often ‘inferior’, they are implicitly endorsing a Han Chinese-centric ethnic dichotomy.

Significantly, the total number of individuals engaged in expressly advancing Han Chinese ethno-nationalism is quite small. However, the number of individuals sympathizing with these perspectives, and visiting online platforms where these views are promoted is notable.^48^ Moreover, those who hold negative views of affirmative action policies, and/or ethnic minorities, could be converted into adopting a more overt form of Han Chinese nationalism.

In sum, Han Chinese identity is historically contingent, drawing meaning from the imagination of a past Han Chinese culture and identity that gained salience in the contemporary era. Furthermore, both minority and majority ethnic identities have been nationalized in the PRC’s ethnic policies, fostering a varying sense of meaning and belonging to both groups; albeit, one that privileges Han Chinese in a variety of important facets.

## Types of Han Chinese privilege

Forms of Han Chinese privilege can be displayed in the labour market and media, and involve state institutions and micro-level everyday interactions between the Han Chinese and the ethnic minority populations. While forms of Han Chinese privilege are illustrated separately later in this article, it should be noted that there is a strong relationship between them. For instance, if ethnic minorities have differential access and benefits in the labour market relative to the Han Chinese, this can potentially lead to a lack of meaningful contact between both groups. The premise being if a Han Chinese earns RMB 5000 a month on average, and an ethnic minority comparatively earns RMB 3000 a month, both groups are most likely to live in varying local geographies; thus, minimizing meaningful sites of physical contact as well as the ability to foster increased social trust between both groups. This, in turn, can serve to perpetuate negative stereotypes and the commodification of ethnic minorities in the media. This can also have spillover effects on the behaviour of institutions to be inclusive; or the behaviour of employers to hire and promote ethnic minorities (whereby trust between employer and employee is a basic component in the recruitment process).

### Labour market

One might reasonably presume that China’s extensive affirmative action policies would benefit ethnic minorities in the labour market, or at least help to mitigate the structural advantages afforded to Han Chinese. However, this has only been partially true. The existing literature on ethnic minority labour market outcomes paints a decidedly mixed portrait. Research into the job application process has found that the Han Chinese have advantages over their ethnic counterparts. Margaret Maurer-Fazio,^49^ performing a resume audit study, found that Han Chinese females were far likelier to receive a job call back than their Mongolian, Tibetan, and Uyghur counterparts.^50^ In a comparable experiment Yue Hou et al.^51^ found significant evidence of discrimination against Muslim-dominated ethnic groups such as Huis and Uyghurs in the hiring process, with this cohort being 50 per cent less likely to receive a response to online job applications; moreover, higher human capital for this cohort of applicants does not fully attenuate this bias.^52^ Similarly, Reza Hasmath and Benjamin Ho^53^ found through interviews that ethnic minorities experienced, or perceived, discrimination on the part of prospective employers at each salient stage of the recruitment process. This was most prevalent in the case of ‘outsider’ ethnic minority groups, those perceived as most dissimilar to Han Chinese culturally and phenotypically such as Turkic ethnic minorities and Tibetans.^54^

After the hiring process, ethnic minorities still report perceptions of discrimination and trouble fitting into the culture of their Han Chinese-dominated workplace.^55^ Xiaogang Wu and Guangye He noted that Uyghurs and Kazakhs, as well as the Hani, Yao, and Dong faced earning wage penalties compared to Han Chinese counterparts in the regions they live.^56^ Such ‘outsider’ ethnic minorities are perceived as less productive and lacking in hard skills, compared to the Han Chinese population.^57^ They face greater discrimination than Han Chinese and/or other ethnic minority populations. Even when outsider ethnic minority groups do have equivalent qualifications, employers may devalue these qualifications since they perceive them as resulting from affirmative action policies. In a study which I conducted,^58^ hiring managers explicitly suggested that although some prospective ethnic minority job candidates were educated in elite universities such as Peking University, the fact that they had ‘assistance’ – via lower scores in the Chinese college entrance examination (高考) – the ‘quality’ of their human capital was not akin to Han Chinese job candidates who did not have the benefit of this assistance. Hou et al. further suggest that employers may prefer to avoid hiring Muslim minorities due to logistical considerations such as the requirement to provide halal food, or the need to accommodate religious prayers, which could be disruptive.^59^ Thus, considering the availability of potential Han Chinese and/or other ethnic minority employees, employers – notably in the private sector – have little incentive to hire outsider ethnic minorities.

It is important to acknowledge that the absence of a wage gap for certain ethnic minorities does not preclude the possibility that an ethnic minority individual could face discrimination from co-workers or employers based on their ethnic status. An example of this can be seen in the Shaoguan toy factory incident. Between May and June 2009, approximately 800 Uyghurs were recruited to work at the Early Light Toy Factory in Shaoguan, Guangdong Province, with tensions quickly emerging between them and their Han Chinese co-workers. Li Qiang, founder of China Labour Watch, a non-governmental organization, claimed that Uyghur employees at the factory faced verbal abuse from hiring managers.^60^ More sinisterly, rumours of sexually aggressive Uyghur men – echoing false historical mythologies by Anglo-Americans towards African-American men^61^ – began to spread among the Han Chinese employees; unfortunately culminating in what was later deemed false accusations that six Uyghur men raped two Han Chinese women.^62^ After the story was initially posted online, a brawl broke out between Han Chinese and Uyghur employees leading to the death of two Uyghurs and 120 injuries – the majority of whom were Uyghurs.^63^ In a far less dramatic example, I tracked the organizational experiences of highly educated, Beijing-born, Tibetans in the capital city for a few years, and the fact that their work life often involved questions about their ‘Tibetan-ness’, their ‘exoticism’ and their ‘foreign-ness’.^64^ Many did not perceive that they fit into the working culture even though they were born and raised in the same location as the majority of their co-workers.

There are a number of interesting analytical questions that emerge when one considers the discussion of labour market discrepancies from a privilege lens. For instance, while a wage gap persists between whites and ethnic minority groups in Western nations,^65^ the wealth gap is far larger and more pervasive.^66^ Although there have been numerous studies on the growing wealth gap in China, the ethnic minority dimension has not been meaningfully explored.^67^ The question as to whether some of the abovementioned advantages afforded to Han Chinese, such as the tendency to dominate in more economically prosperous regions or possessing more developed social networks that can lead to greater economic opportunities, is a major component of Han Chinese privilege worth examining.

### Media

An often-discussed ethno-racial privilege is the way the privileged and non-privileged groups are portrayed in the media. For example, across all forms of media, non-privileged groups can often be presented in a stereotypical fashion, not as fully formed individuals but rather as narrowly constructed tropes. For instance, according to Qin Zhang, Asian women in Western contexts are portrayed as ‘silent, humble, obedient, exotic, and hypersexualized dolls, or as evil, deceitful, seductive, and ruthless dragon ladies’.^68^ These stereotypes are by no means static and are constructed and reconstructed based on the sociocultural environment of the time.

The Chinese context is not dissimilar. Ethnic minority populations are often depicted in the media in a highly stereotyped and commodified fashion. For example, Louisa Schein found that ethnic minority women, especially those from south China, are portrayed as either innocent, backward ‘colourful flowers among the natural flora and fauna’ or ‘as very human objects of erotic fascination. In many images they appeared voluptuous, more extensively revealed than would be proper for a Han Chinese woman’.^69^ The depiction of ethnic minorities as feminine, backward, and exotic is common in media portrayals, and is often contrasted against the Han Chinese who are masculine, industrious, and modern.^70^

An analysis of the ‘Han Clothing Movement’ came to similar conclusions.^71^ Han Chinese-ness is portrayed as ‘positive, progressive and humane’; and non-Han Chinese-ness the opposite.^72^ This ultimately reproduces and reaffirms specific power relations that place Han Chinese as the centre of power relations and reinforces Han Chinese privilege in the everyday.

Emblematic of this is the portrayal of ethnic minorities in the opening ceremony of the 2008 Beijing Olympics. The 55 ethnic minority groups, portrayed largely by Han Chinese performers, were dressed in traditional colourful, cultural costumes; in contrast, the Han Chinese group was portrayed wearing Western-style suits and dresses – symbols of sophistication and modernity in China. The contrast between the colourful ‘ethnic minorities’ and the Western-style garbed Han Chinese further enhances the perception of ethnic minorities as exotic, foreign, and backward. This dichotomous representation is also seen amongst Uyghurs who face unique discriminatory depictions as ‘dangerous’, and ‘threatening’, which is akin to depictions of Muslims in many Western jurisdictions.^73^

Interestingly, not all minority populations are subject to the same degree of commodification and stereotyping in the media. In another study that I conducted, highly educated and more integrated populations such as the ethnic minority Koreans are presented in a more balanced manner on popular variety shows.^74^ One way of interpreting this is that these groups are harder to negatively stereotype as backward, considering their economic and educational successes, and are therefore seen as more akin to the Han Chinese population.

Social media is not immune from negative stereotyping and harassment of China’s ethnic minorities. This is especially true for the nation’s Muslim ethnic minority groups. Luwei Luqiu and Fan Yang found that the state’s official atheistic ideology influenced negative comments towards Muslims, with netizens engaged in this behaviour often suggesting that Chinese Muslims were an outgroup who were ‘weak’, ‘superstitious’, and even ‘non-conforming’ because of their faith.^75^ Stereotyping and criticism of ethnic minority populations by Han Chinese netizens often draw upon historical justifications and claims that the Han Chinese are the victim group.^76^ For instance, the Tongzhi Hui Rebellion is invoked to suggest that the Hui cannot be trusted, and that the Mongol-ruled Yuan dynasty and Manchu-ruled Qing dynasty were barbarians who destroyed Han civilization, and imposed their violent rule upon the Han Chinese people.^77^

Furthermore, as previously discussed, preferential treatment afforded to minority populations often inflames rancour among members of the Han Chinese majority who disagree with the state providing benefits and resources to the ethnic minority populations. For some Han Chinese ethno-nationalists, the weakness of Han Chinese identity, coupled with existing affirmative action programmes for ethnic minorities, are taken together to suggest that the Han Chinese are the ones facing the most discrimination.^78^ This is comparable to the narrative amongst some white nationalists in Western nations.

### Other potential manifestations

Research into other potential manifestations of Han Chinese privilege is still relatively nascent. However, there is evidence to suggest that the Han Chinese population disproportionately benefit from broader public institutions. For example, in the field of education, Han Chinese knowledge, history, and culture are privileged, whereas ethnic minorities’ history, knowledge, and culture are severely under-represented.^79^ The miniscule number of textbooks that discuss ethnic minority populations has a tendency to present a very narrow picture of ethnic identity. As Yuxiang Wang and JoAnn Phillion found in their study of elementary school textbooks that had minority content:

Minority festivals, clothing, and customs are the focus. Minority heroes, history, literature, and music are excluded from said textbooks. Minority issues related to language, culture, identity, poverty, minority students’ high dropout rates, and poor economic development are omitted.^80^

Similar to observations pertaining to the media, in many of these texts Han Chinese knowledge and consequently, the Han Chinese, appear as civilized in contrast to depictions of the backward ethnic minorities.^81^ This parallels much of the treatment of Indigenous peoples’ culture, history, and knowledge in settler societies such as Australia, Canada, and the United States.

Other areas where there have been some indications of Han Chinese privilege include access to health care,^82^ differential treatment in the justice and policing system,^83^ access to external finance,^84^ and access to government connections;^85^ the latter two points being important components for starting and succeeding in the private sector.

## The absence of Han privilege discourse

Han Chinese enjoy privilege across a range of contexts, not dissimilar to those that have been documented for whites in multi-ethnic Western societies. However, unlike many jurisdictions where discussion of ethno-racial privilege has become commonplace, not only in academia, but in politics, media, and everyday conversations, this has not meaningfully occurred in China. There are several primary reasons for this.

First, official state policies and philosophy conceive a unified Chinese nation comprising 56 ethnic groups who live in a ‘harmonious’ situation. The CCP has carefully managed any discussions that officially divert from this standpoint. This can be seen in the framing of affirmative action policies as being necessary for modernizing the underdeveloped, or even backward, ethnic minority populations. From the 1950s onwards, this was portrayed as the older brother (Han Chinese) helping the younger brothers (ethnic minorities) to achieve a higher state of progress from a Marxian perspective.^86^

While the state has allowed minimal criticism of these policies to publicly circulate, they are usually conducted by members of the privileged, Han Chinese community, for example the writings of Ma Rong, rather than the non-privileged, ethnic minority community. The type of large-scale mobilization of non-privileged ethnic minority groups around (perceived or actual) structural inequalities witnessed in multi-ethnic Western nations, such as the Civil Rights Movement or the anti-police brutality protests, would be unacceptable to the CCP. There are good reasons for this from the CCP’s perspective – notably when considering the role that ethnic tensions played in the collapse of relatively similar political regimes to the PRC such as the former Yugoslavia and the Soviet Union.

Second, the prevalent conception that Han Chinese have of their own identity is vastly different to the perception that whites in Western jurisdictions have of themselves. For instance, awareness around the role of whites in the slave trade, colonization, and other mistreatment of non-whites are relatively well known and taught in Western nations. Therefore, for some whites there is a recognition, and even a sense of guilt, for the sufferings that whites have inflicted upon non-whites. Conversely, in China there is a strong focus on the ‘century of humiliation’ that the nation suffered at the hands of Western powers and Japan. As such, a perception exists that China itself, and the Han Chinese in particular, are victims, rather than being the bearers of any form of privilege.^87^

Compounding this is the frequent discussion in Chinese traditional and social media, of the legitimate instances of discrimination and prejudice that Chinese nationals and immigrants face abroad.^88^ In addition, as previously mentioned, the affirmative action policies that have been implemented in China have generated anger among some Han Chinese netizens. This has failed to promote inter-ethnic harmony, and has rather ‘led part of the Han Chinese population to ignore the current structural inequality that ethnic minorities face’.^89^

Simply put, Han Chinese privilege has not become a salient concept since many Han Chinese do not see themselves as privileged vis-a-vis China’s ethnic minorities. There is, of course, a degree of truth to this if one looks at the experiences of some successful ethnic minority groups in the labour market such as the Koreans. Moreover, there are some Han Chinese nationalists who see the celebrations of ethnic minority culture as problematic considering that Han Chinese cultural symbols such as dances, songs, and clothes do not receive the same attention.^90^

## Conclusion

Han Chinese privilege and white privilege share several commonalities in their operations and performance. They both emerged from drawing distinctions between the ethnic majority group and the Other in society. Such ethnic majority identities gained new meaning when examining the privileges afforded to them in the labour market and popular media; with state policies being instrumental in this process.

There are notable differences in the operation of Han Chinese privilege and white privilege. For instance, phenotypical difference is the common mechanism for ascribing white identity and, consequently, privilege. In the Han Chinese case, however, although phenotypical differences can play a role – especially for visibly different ethnic minority populations such as the Turkic populations and Tibetans – state ethnic policies play an even greater role. For example, the fixed ethnic categorization system has served to give new meaning to both ethnic identity and Han Chinese identity through the dissatisfaction that some Han Chinese harbour towards state preferential policies for ethnic minorities. These policies have served as a double-edged sword by not only improving the economic conditions of ethnic minorities, but also stoking resentment among the majority, Han Chinese population.

Notwithstanding the absence of a Han Chinese privilege discourse, there are pragmatic reasons why Han Chinese privilege can become a more salient concept in the foreseeable future. There is growing evidence to suggest that the CCP is listening to Han Chinese grievances and is planning to remove preferential policies for all ethnic minorities.^91^ If such reforms are done wholesale, without factoring the socio-economically vulnerable ethnic minority groups (e.g. ‘outsider’ minorities), this could accelerate the saliency of ethnicity across the PRC, increase ethno-nationalism, and bring to the forefront the notion of Han Chinese privilege.

## Notes

1. Reza Hasmath (ed.), *Managing Ethnic Diversity: Meanings and Practices from an International Perspective*, Burlington, VT: Ashgate, 2011.

2. Reza Hasmath, What salience does white privilege have in non-diverse societies? (paper presented at the American Sociological Association Annual Meeting, virtual, 6–10 August 2021).

3. Junxi Quan and Hong Zhu, Han Chinese ‘drifters’ in Lhasa: The ambivalent cultural politics of Tibetanness amidst China’s geographies of modernity, *Social and Cultural Geography* 17(7), 2016: 892–912; Joanne N. Smith, ‘Making culture matter’: Symbolic, spatial and social boundaries between Uyghurs and Han Chinese, *Asian Ethnicity* 3(2), 2002: 153–74; and David R. Stroup, Han–Hui relations and Chinese regime legitimation in the Xi Jinping era: ‘God is a drug’, *Asian Survey* 60(4), 2020: 659–84.

4. National Bureau of Statistics, Main data of the Seventh National Population Census news release, 11 May 2021, http://www.stats.gov.cn/english/PressRelease/202105/t20210510_1817185.html, accessed 26 May 2023.

5. Dru C. Gladney, *Dislocating China: Muslims, Minorities, and Other Subaltern Subjects*, Chicago: University of Chicago Press, 2004; Louisa Schein, Gender and internal orientalism in China, *Modern China* 23(1), 1997: 69–98.

6. Stevan Harrell, *Ways of Being Ethnic in Southwest China*, Seattle: University of Washington Press, 2001, 295.

7. James Leibold, More than a category: Han supremacism on the Chinese Internet, *China Quarterly* 203, 2010: 539–59.

8. Ruth Frankenberg, *White Women, Race Matters: The Social Construction of Whiteness*, Minneapolis: University of Minnesota Press, 1993.

9. Reza Hasmath, Discourse, deliberation and difference in an authoritarian public sphere, *Journal of Deliberative Democracy* 19(1): 1–10.

10. Graziella Moraes Silva, Luciana Souza Leão, and Barbara Grillo, Seeing whites: Views of black Brazilians in Rio de Janeiro, *Ethnic and Racial Studies* 43(4), 2020: 632–51.

11. Jon E. Fox, Laura Moroşanu, and Eszter Szilassy, The racialization of the new European migration to the UK, *Sociology* 46(4), 2012: 680–95; Noel Ignatiev, *How the Irish Became White*, New York: Routledge, 2009.

12. John Hartigan Jr., *Racial Situation: Class Predicaments of Whiteness in Detroit*, Princeton: Princeton University Press, 1999.

13. Jacqueline A. Housel, Geographies of whiteness: The active construction of racialized privilege in Buffalo, New York, *Social and Cultural Geography* 10(2), 2009: 131–51, 134.

14. Peggy McIntosh, White privilege and male privilege: A personal account of coming to see correspondences through work in women’s studies, in Margaret L. Andersen and Patricia Hill Collins (eds) *Race, Class, and Gender: An Anthology*, Belmont, CA: Wadsworth, 1988, 76–87.

15. David R. Roediger, *The Wages of Whiteness: Race and the Making of the American Working Class*, London: Verso, 2007.

16. Teresa J. Guess, The social construction of whiteness: Racism by intent, racism by consequence, *Critical Sociology* 32(4), 2006: 649–73.

17. Hasmath, What salience does white privilege have in non-diverse societies?

18. Zeus Leonardo, The color of supremacy: Beyond the discourse of ‘white privilege’, *Educational Philosophy and Theory* 36(2), 2004: 137–52, 139.

19. Anne Bonds and Joshua Inwood, Beyond white privilege: Geographies of white supremacy and settler colonialism, *Progress in Human Geography* 40(6), 2016: 715–33.

20. Ibid., 716.

21. Leibold, More than a category, 542.

22. For further discussion, see Magnus Fiskesjӧ, Rescuing the empire: Chinese nation-building in the twentieth century, *European Journal of East Asian Studies* 5(1), 2006: 15–44.

23. Huang Xingtao 黄兴涛, 重塑中华: 近代中国「中华民族」观念研究 (Reshaping China: A study on the concept of the ‘Chinese nation’ in modern China), Beijing: 北京师范大学出版社 (Beijing Normal University press), 2017.

24. Ignatiev, *How the Irish Became White*.

25. Margaret Maurer-Fazio and Reza Hasmath, The contemporary ethnic minority in China: An introduction, *Eurasian Geography and Economics* 56(1), 2015: 1–7.

26. For further discussion, see Xiaowei Zang, *Ethnicity in China: A Critical Introduction*, Cambridge, UK: Polity Press, 2015.

27. Reza Hasmath, *A Comparative Study of Minority Development in China and Canada*, New York: Palgrave Macmillan, 2010.

28. Xiaoyuan Liu, *Frontier Passages: Ethnopolitics and the Rise of Chinese Communism, 1921–1945*, Washington, DC and Palo Alto, CA: Woodrow Wilson Center Press and Stanford University Press, 2003.

29. Gladney, *Dislocating China*.

30. Hasmath, *A Comparative Study of Minority Development in China and Canada*.

31. Leibold, More than a category.

32. Mao Zedong, Criticize Han chauvinism, *Chinese Law and Government* 14, 1981: 53–4.

33. Zhou Enlai, *Policy Towards Nationalities of the People’s Republic of China*, Beijing: Foreign Languages Press, 1953, 25.

34. Hasmath, *A Comparative Study of Minority Development in China and Canada*.

35. Gladney, *Dislocating China*.

36. Reza Hasmath, The education of ethnic minorities in Beijing, *Ethnic and Racial Studies* 34(11), 2011: 1835–54; Reza Hasmath and Andrew W. MacDonald, Beyond special privileges: The discretionary treatment of ethnic minorities in China’s welfare system, *Journal of Social Policy* 47(2), 2018: 295–316.

37. Ibid.

38. (Still) mostly Han men: Demographics of the 14th NPC, NPC Observer, 25 February 2023, https://npcobserver.com/2023/02/25/china-14th-npc-demographics/, accessed 19 July 2023.

39. Reza Hasmath, Future responses to managing Muslim ethnic minorities in China: Lessons learned from global approaches to improving inter-ethnic relations, *International Journal* 77(1), 2022: 51–67.

40. Björn A. Gustafsson, Reza Hasmath, and Sai Ding (eds), *Ethnicity and Inequality in China*, New York: Routledge, 2021.

41. Reza Hasmath, The interactions of ethnic minorities in Beijing, *Centre on Migration, Policy and Society Working Paper* 14-111, 2014, https://www.compas.ox.ac.uk/2014/wp-2014-111-hasmath_ethnic_minorities_beijing/#:∼:text=The%20Interactions%20of%20Ethnic%20Minorities%20in%20Beijing%20Reza,Download%20WP-2014-111-Hasmath_Ethnic_Minorities_Beijing%20%28PDF%29%20...%203%20Topics%204%20Regions, accessed 3 August 2023.

42. There is no classification of mixed ethnic identity in China. Parents of children born from inter-ethnic marriages select one of their ethnicities for the offspring. From age 18, they have a two-year window to change their ethnicity to the other parent’s ethnic identity.

43. In practice, ethnic minority re-registration generally occurred amongst particular ethnic minority groups such as Manchus, who had suffered censure as former rulers during the last imperial (Qing) dynasty.

44. It should be acknowledged that during the population years cited, the one-child per-couple policy was intact, whereby ethnic minorities were, in general, exempt. This could potentially skew the figures noted.

45. Mimi Lau, From Xinjiang to Ningxia, China’s ethnic groups face end to affirmative action in education, taxes, policing, *South China Morning Post*, 5 December 2019, https://www.scmp.com/news/china/politics/article/3040577/chinas-ethnic-groups-face-end-affirmative-action-education, accessed 26 May 2023.

46. Ma Rong 马戎, 当前中国民族问题研究的选题与思路 (Topics of and thoughts on the present-day ethnic studies in China), 中央民族大学学报(哲学社会科学版) (Journal of Minzu University of China (philosophy and social sciences edition)), no. 3, 2007: 12–38.

47. Luwei Rose Luqiu and Fan Yang, Islamophobia in China: News coverage, stereotypes, and Chinese Muslims’ perceptions of themselves and Islam, *Asian Journal of Communication* 28(6), 2018: 598–619; Luwei Rose Luqiu and Fan Yang, Anti-Muslim sentiment on social media in China and Chinese Muslims’ reactions to hatred and misunderstanding, *Chinese Journal of Communication* 13(3), 2020: 258–74.

48. Leibold, More than a category.

49. Margaret Maurer-Fazio, Ethnic discrimination in China’s Internet job board labor market, *IZA Journal of Migration* 1, 2012, https://link.springer.com/article/10.1186/2193-9039-1-12, accessed 10 July 2023.

50. Interestingly enough, ethnic minority women were less disadvantaged in the labour market than Han women. This was largely due to smaller wage penalties linked to marriage and having children. See Hua-Yu Sebastian Cherng, Reza Hasmath, and Benjamin Ho, Holding up half the sky? Ethno-gender labour market outcomes in China, *Journal of Contemporary China* 28(117), 2019: 415–33.

51. Yue Hou, Chuyu Liu, and Charles Crabtree, Anti-Muslim bias in the Chinese labor market, *Journal of Comparative Economics* 48(2), 2020: 235–50.

52. Lower returns to education for Uyghurs was also reported in Reza Hasmath, What explains the rise of majority–minority tensions and conflict in Xinjiang?, *Central Asian Survey* 38(1), 2019: 46–60.

53. Reza Hasmath and Benjamin Ho, Job acquisition, retention, and outcomes for ethnic minorities in urban China, *Eurasian Geography and Economics* 56(1), 2015: 24–43.

54. Andrew W. MacDonald and Reza Hasmath, Outsider ethnic minorities and wage determination in China, *International Labour Review* 158(3), 2019: 489–508.

55. Reza Hasmath, From job search to hiring and promotion: The labour market experiences of ethnic minorities in Beijing, *International Labour Review* 150(1–2), 2011: 189–201.

56. Xiaogang Wu and Guangye He, Changing ethnic stratification in contemporary China, *Journal of Contemporary China* 25(102), 2016: 938–54.

57. Hou, Liu, and Crabtree, Anti-Muslim bias in the Chinese labor market.

58. Hasmath, From job search to hiring and promotion.

59. Hou, Liu, and Crabtree, Anti-Muslim bias in the Chinese labor market.

60. Andrew Jacobs, At a factory, the spark for China’s violence, *New York Times*, 16 July 2009, https://www.nytimes.com/2009/07/16/world/asia/16china.html, accessed 26 May 2023.

61. See for example Winthrop D. Jordan, *White over Black: American Attitudes Toward the Negro, 1550–1812*, Chapel Hill: University of North Carolina Press, 2012.

62. Johnathan Watts, Old suspicions magnified mistrust into ethnic riots in Urumqi, *The Guardian*, 10 July 2009, https://www.theguardian.com/world/2009/jul/10/china-riots-uighurs-han-urumqi, accessed 26 May 2023.

63. Jacobs, At a factory, the spark for China’s violence.

64. Reza Hasmath, The big payoff? Educational and occupational attainments of ethnic minorities in Beijing, *European Journal of Development Research* 20(1), 2008: 104–16.

65. Reza Hasmath, *The Ethnic Penalty: Immigration, Education and the Labour Market*, Burlington, VT: Ashgate, 2012.

66. Kriston McIntosh et al., Examining the black–white wealth gap, Brookings Institute, 2020, https://www.brookings.edu/blog/up-front/2020/02/27/examining-the-black-white-wealth- gap/, accessed 26 May 2023.

67. Shi Li and Renwei Zhao, Changes in the distribution of wealth in China, 1995–2002, in James Davies (ed.) *Personal Wealth from a Global Perspective*, Oxford: Oxford University Press, 2008, 93–111; Thomas Piketty, Li Yang, and Gabriel Zucman, Income inequality is growing fast in China and making it look more like the US, LSE Business Review, 1 April 2019, https://blogs.lse.ac.uk/businessreview/2019/04/01/income-inequality-is-growing-fast-in-china-and-making-it-look-more-like-the-us/, accessed 26 May 2023; and Yu Xie and Yongai Jin, Household wealth in China, *Chinese Sociological Review* 47(3), 2015: 203–29.

68. Qin Zhang, Asian Americans beyond the model minority stereotype: The nerdy and the left out, *Journal of International and Intercultural Communication* 3(1), 2010: 20–37, 20.

69. Schein, Gender and internal orientalism in China, 77.

70. Matthew Hoddie, Minorities in the official media: Determinants of state attention to ethnic minorities in the People’s Republic of China, *International Journal of Press/Politics* 11(4), 2006: 3–21; Schein, Gender and internal orientalism in China, 77. It should be acknowledged that ethnic minorities can have agency to construct their ethnic identities in contradistinction to the majority ethnic group, Han Chinese, see for example Ben Hillman and Lee-Anne Henfry, Macho minority: Masculinity and ethnicity on the edge of Tibet, *Modern China* 32(2), 2006: 251–72.

71. See for example Kevin Carrico, *The Great Han: Race, Nationalism, and Tradition in China Today*, Berkeley: University of California Press, 2017.

72. Yinghong Cheng. *Discourses of Race and Rising China*, New York: Palgrave Macmillan, 2019.

73. Sean R. Roberts, The biopolitics of China’s ‘war on terror’ and the exclusion of the Uyghurs, *Critical Asian Studies* 50(2), 2018: 232–58.

74. Hasmath, The interactions of ethnic minorities in Beijing.

75. Luqiu and Yang, Islamophobia in China.

76. Leibold, More than a category.

77. Luqiu and Yang, Islamophobia in China.

78. Lau, From Xinjiang to Ningxia.

79. Yitin Chu, Constructing *minzu*: The representation of *minzu* and *Zhonghua Minzu* in Chinese elementary textbooks, *Discourse* 39(6), 2018: 941–53; Dong Zhao, Religious identity and cultural refashioning: Educational constraints for migrant Muslim Hui university students in China, *Diaspora, Indigenous, and Minority Education* 8(2), 2014: 59–74.

80. Yuxiang Wang and JoAnn Phillion, Whose knowledge is valued: A critical study of knowledge in elementary school textbooks in China, *Intercultural Education* 21(6), 2010: 567–80.

81. Ibid., 572.

82. Amanda Harris et al., Challenges to maternal health care utilization among ethnic minority women in a resource-poor region of Sichuan Province, China, *Health Policy and Planning* 25(4), 2010: 311–18.

83. Magnus Fiskesjӧ, State strategies to implement (and hide) genocide in China and Myanmar, since 2017, in David J. Simon and Leora Kahn (eds) *Handbook of Genocide Studies*, Cheltenham, UK: Edward Elgar, 2023, 123–41.

84. Anthony Howell, Ethnic entrepreneurship, initial financing, and business performance in China, *Small Business Economics* 52(3), 2019: 697–712.

85. Tyler Harlan, Fringe existence: Uyghur entrepreneurs and ethnic relations in urban Xinjiang, in Ben Hillman and Gray Tuttle (eds) *Ethnic Conflict and Protest in Tibet and Xinjiang: Unrest in China’s West*, New York: Columbia University Press, 2016, 179–200.

86. David Y. H. Wu, Chinese minority policy and the meaning of minority culture: The example of Bai in Yunnan, China, *Human Organization* 49(1), 1990: 1–13.

87. Orville Schell and John Delury, *Wealth and Power: China’s Long March to the Twenty-First Century*, New York: Random House, 2013.

88. Xenophobia against Asians over coronavirus rings alarm bells in U.S., 12 February 2020, http://english.cctv.com/2020/02/12/ARTILlv0rYlAazVCffp4U7mK200212.shtml, accessed 26 May 2023.

89. Luqiu and Yang, Islamophobia in China, 9.

90. Leibold, More than a category.

91. Masahiro Hoshino, Preferential policies for China’s ethnic minorities at a crossroads, *Journal of Contemporary East Asia Studies* 8(1), 2019: 1–13.
